# Traffic-Related Air Pollution and Ground-Level Ozone Associated Global DNA Hypomethylation and Bulky DNA Adduct Formation

**DOI:** 10.3390/ijms24032041

**Published:** 2023-01-20

**Authors:** Armelle Munnia, Valentina Bollati, Valentina Russo, Luca Ferrari, Marcello Ceppi, Marco Bruzzone, Stefano Dugheri, Giulio Arcangeli, Franco Merlo, Marco Peluso

**Affiliations:** 1 Research Branch, Regional Cancer Prevention Laboratory, ISPRO-Study, Prevention and Oncology Network Institute, 50139 Florence, Italy; 2 EPIGET Department of Clinical Sciences and Community Health, Università Degli Studi di Milano, 20122 Milan, Italy; 3 Occupational Health Unit, Fondazione IRCCS Ca’ Granda Ospedale Maggiore Policlinico, 20122 Milan, Italy; 4 Clinical Epidemiology Unit, IRCCS Ospedale Policlinico San Martino, 16132 Genoa, Italy; 5 Laboratorio di Igiene e Tossicologia Industriale, Azienda Ospedaliero-Universitaria Careggi, 50134 Florence, Italy; 6 Dipartimento di Medicina Sperimentale e Clinica, Università degli Studi di Firenze, 50121 Florence, Italy; 7 Research and Statistics Infrastructure, Azienda Unità Sanitaria Locale, IRCCS, 42121 Reggio Emilie, Italy

**Keywords:** air pollution, ozone, DNA adducts, *ALU*, *IL-6*

## Abstract

Studies have indicated that air pollution, including surface-level ozone (O_3_), can significantly influence the risk of chronic diseases. To better understand the carcinogenic mechanisms of air pollutants and identify predictive disease biomarkers, we examined the association between traffic-related pollutants with DNA methylation alterations and bulky DNA adducts, two biomarkers of carcinogen exposure and cancer risk, in the peripheral blood of 140 volunteers—95 traffic police officers, and 45 unexposed subjects. The DNA methylation and adduct measurements were performed by bisulfite-PCR and pyrosequencing and ^32^P-postlabeling assay. Airborne levels of benzo(a)pyrene [B(a)P], carbon monoxide, and tropospheric O_3_ were determined by personal exposure biomonitoring or by fixed monitoring stations. Overall, air pollution exposure was associated with a significant reduction (1.41 units) in global DNA methylation (95% C.I. −2.65–0.04, *p* = 0.026). The decrement in *ALU* repetitive elements was greatest in the policemen working downtown (95% C.I. −3.23–−0.49, *p* = 0.008). The DNA adducts were found to be significantly increased (0.45 units) in the municipal officers with respect to unexposed subjects (95% C.I. 0.02–0.88, *p* = 0.039), mainly in those who were controlling traffic in downtown areas (95% C.I. 0.39–1.29, *p* < 0.001). Regression models indicated an increment of *ALU* methylation at higher B(a)P concentrations (95% C.I. 0.03–0.60, *p* = 0.032). Moreover, statistical models showed a decrement in *ALU* methylation and an increment of DNA damage only above the cut-off value of 30 µg/m^3^ O_3_. A significant increment of 0.73 units of *IL-6* gene methylation was also found in smokers with respect to non-smokers. Our results highlighted the role of air pollution on epigenetic alterations and genotoxic effects, especially above the target value of 30 µg/m^3^ surface-level O_3_, supporting the necessity for developing public health strategies aimed to reduce traffic-related air pollution molecular alterations.

## 1. Introduction

Air quality is a recognized health problem in metropolitan areas, owing to potential carcinogen exposure to workers and nearby residents from air pollution from urban traffic. Air pollution is considered to be a causative agent for cardiovascular, respiratory, and cancer diseases [1], contributing to about seven million premature deaths per year in the world [2]. It consists of complex volatile mixtures of gases and solid particulate matter, including carbonaceous particles associated with adsorbed organic chemicals, such as polycyclic aromatic hydrocarbons (PAHs) and ozone (O_3_). The polycyclic aromatic hydrocarbons are environmental contaminants derived from the incomplete combustion of organic materials and are present in motor vehicle exhausts [3], whereas ground-level O_3_ is formed by chemical reactions of oxides of nitrogen and volatile organic compounds in the presence of sunlight [4]. 

The PAHs are a recognized class of carcinogens [3], that can be metabolically activated by cytochrome P450 monooxygenases into reactive electrophiles able of interacting with DNA forming bulky adducts [5]. The DNA adducts, if not eliminated by DNA repair machinery, can induce mutations initiating the sequential process of carcinogenesis [6]. They are considered to be a reliable biomarker of carcinogen exposure [7,8] and cancer risk [9,10]. Recently, we indicated that the generation of DNA damage at single nucleotide resolution causes characteristic signatures at the site of mutations along the sequence of the *tumor suppressor gene TP53* (*TP53*), indicating a causal relationship between DNA damage and human cancer [11,12]. In addition to genotoxic effects, air pollution can cause various epigenetic alterations, as on the levels of DNA methylation and non-coding RNA transcripts [1], important regulators for gene transcription [13]. Changes in DNA methylation describe the attachment of methyl groups to DNA, usually at the 5’ position of the cytosine ring, leading to the formation of 5-methylcytosine (5-mC), including at CpG sites, by the action of DNA methyltransferases. The DNA methylation is an epigenetic mechanism that contributes to suppressing gene expression and maintaining genome stability and its alteration is a frequent characteristic of a wide variety of chronic diseases [14]. Today, it is widely accepted that the inactivation of tumour-suppressor genes can occur as a consequence of hypermethylation in the promoter domains, whereas hypomethylation of repetitive elements can cause genome instability and inappropriate activation of oncogenes [14]. Further, a number of epigenetic studies showed that air pollution exposure can result in a general decrement in DNA methylation [1], including in genes related to inflammation and immunity [15,16]. Specific traffic-related air pollutants, including particulate matter, O_3_, and PAHs, have also been associated with changes in DNA methylation levels [1]. Prenatal exposure to air pollution has been even linked to differential methylation patterns [1]. 

All these investigations emphasize the effects of toxic air pollutant mixtures on the epigenome and genome; however, many adverse effects remain unclear and other efforts are needed to improve the understanding of the effects on epigenome and genome. Therefore, we examined the associations between traffic-related air pollution and its components with changes in global and gene-specific DNA methylation marks and with the generation of bulky DNA adducts in an observational study conducted on a cohort of traffic policemen in Genoa, Italy. The measurements of DNA methylation in the repetitive elements of *LINE-1* and *ALU* and the promoter domains of the *inducible nitric oxide synthase* (i*NOS*) and the *IL-6* genes in the peripheral white blood cells (WBCs) of study participants were performed by bisulfite-PCR and pyrosequencing [17], whereas the formation of bulky DNA adducts was analysed blindly by the ^32^P-postlabeling technique [18]. Airborne concentrations of benzo(a)pyrene [B(a)P] and carbon monoxide (CO) were obtained by personal exposure monitoring and the ground-level O_3_ by fixed monitoring stations. Our main aim was to evaluate environmental challenges induced by air pollution, including low tropospheric O_3_. Increasing knowledge of how exposure to air pollutants can induce different molecular alterations can impact the risk assessment of chemical carcinogens and can be useful to develop preventative and remedial strategies aimed to reduce morbidity in polluted environments in Western countries.

## 2. Results 

### 2.1. Demographic Variables

The present observational study involved 140 subjects, 35.7 ± 5.3 (SD) years of age. There were 95 traffic police officers, 35.6 ± 4.9 (SD) years of age, 36% were smokers; and 45 participants were unexposed subjects, 35.8 ± 5.9 (SD) years of age, 36% smokers. Police officers were also classified by three working districts, e.g., seaside, hill, and downtown.

### 2.2. Air Pollution Biomonitoring

Distributions of the average levels of B(a)P, CO, and O_3_ are reported in Table 1. The levels and range of B(a)P ng/m^3^ in the city air of Genoa, Italy were 4.2 ± 2.6 (SD), 0.5–13.9 ng/m^3^ among the traffic policemen, and 0.2 ± 0.3 (SD), 0.03–1.1 ng/m^3^ in unexposed subjects. The mean concentrations and range of CO mg/m^3^ in the city atmosphere of Genoa, Italy were 8.7 ± 4.2 (SD), 1–28 mg/m^3^ among the municipal policemen and 1.1 ± 0.4 (SD), range 1–13, in unexposed subjects. The daily mean concentrations and range of O_3_ µg/m^3^ in the city air of Genoa, Italy were 44.2 ± 23.5 (SD), 11–101 µg/m^3^. Significantly higher levels of B(a)P and CO pollutants were found by personal exposure biomonitoring in the traffic policemen with respect to unexposed subjects (*p* < 0.001). The concentrations of B(a)P and CO in the city atmosphere of Genoa, Italy were not statistically different among the three different districts (i.e., seaside, hill, and city centre suburbs). Conversely, significantly higher levels of tropospheric O_3_ were revealed in the city air of the hill and downtown districts of Genoa, Italy with respect to seaside suburbs (*p* = <0.001 and *p* = 0.003, respectively). 

### 2.3. Air Pollution and Epigenetic Marks

The frequencies of epigenetic damage marks are reported in Table 2. Results show a decrement in global methylation rate in the *ALU* repetitive elements of the traffic policemen with respect to unexposed subjects, 29.3 ± 2.7 (SD) vs. 31.1 ± 2.7 (SD). The greatest change was detected in those who were working in downtown, 28.8 ± 2.9 (SD). The level of DNA methylation in the promoter domain of the *IL-6* gene was found to be higher in smokers as compared to non-smokers.

Multivariable regression models, including age, sex, and smoking habit, as independent variables, were used to compute the differences in the means of epigenetic marks between traffic policemen and unexposed subjects, as reported in Table 3. Regression models estimated a significant reduction of 1.41 units in *ALU* methylation in the municipal policemen with respect to unexposed subjects (95% C.I. −2.65–0.04, *p* = 0.026). A decrement of 1.86 units was even found in the downtown police officers (95% C.I. −3.23–−0.49, *p* = 0.008). A significant increment of 0.73 units of *IL-6* methylation was observed in smokers as compared to non-smokers.

Regression models, adjusted by sex, age, smoking habit, and occupational exposure, were employed to examine the differences in the means of epigenetic marks considering specific components of air pollution (Table 4). Multivariable regression models showed a significant increment of global methylation in the *ALU* repetitive elements at higher levels of B(a)P. Specifically, when the external concentrations of B(a)P increased by one unit, the rate of global methylation in *ALU* was significantly enhanced by 0.32 units on average (95% C.I. 0.03–0.60, *p* = 0.032). When we explored the levels of epigenetic aberrations caused by exposure to tropospheric O_3_ at low exposure levels, a significant ALU methylation reduction was observed only at tropospheric O_3_ above the cut-off value of 30 µg/m^3^ O_3_. The rate of *ALU* methylation was significantly reduced by 1.65 units (95% C.I. −3.01–−0.30, *p* = 0.017) for concentrations of O_3_ above the cut-off value.

### 2.4. Air Pollution and Bulky DNA Adducts

A typical diagonal radioactive zone chromatographic pattern with several adduct-spots was generally detected in the chromatograms of police officers with respect to those of unexposed control. The visual investigation of the two adduct standards revealed a major spot, possibly caused by the N^2^ guanine substitution by *anti*-b(*a*)p 7,8-dihydrodiol 9,10-oxide (*anti*-BPDE). The levels of bulky DNA adducts were 103 ± 67 (SD) per 10^8^ normal nucleotides in the BPDE-treated calf-thymus DNA and 19 ± 9 (SD) adducts per 10^8^ normal nucleotides in the livers of B(a)P-treated experimental animals, respectively, by ^32^P-postlabeling. Comparable adduct levels, e.g., 20 adducts per 10^8^ normal nucleotides, were detected in the hepatic tissues of the livers of B(a)P-treated experimental animals by GC-MS [19]. The levels of bulky DNA adducts are reported in Table 3. 

Overall, we found that air pollution exposure was correlated with a higher level of different types of bulky DNA adducts, such as benzo(a)pyrene, lactone, and quinone-adducts and bulky oxidative lesions [4,20], in adjusted models. A greater generation of bulky DNA adducts was found in the police officers with respect to unexposed subjects, with the highest increment in the city centre, whereas no significant effect of smoking habit with DNA damage was observed (Table 4). In detail, the production of bulky DNA adducts was significantly different in the police officers with respect to unexposed subjects (95% C.I. 0.02–0.88, *p* = 0.039), especially in those who were working in the city centre (95% C.I. 0.39–1.29, *p* < 0.001). Conversely, no significant association with DNA damage was detected. Finally, when the associations with specific pollutants and the cut-off value of 30 µg/m^3^ O_3_ were considered, only a significant increment of the production of bulky DNA adducts of 0.56 units (95% C.I. 0.09–1.03, *p* = 0.019) was found above the target value of 30 µg/m^3^ O_3_ (Table 5).

## 3. Discussion

Biomarkers were introduced in environmental epidemiology under the assumption that they could improve the assessment of health effects caused by carcinogen exposure. One of the main advantages is that surrogate biomarkers can be used in identifying and understanding the mechanisms of specific airborne components that can be linked to a high risk of chronic diseases, including human cancer [21]. Therefore, evaluating the relationships between traffic-related air pollutants and epigenetic and DNA damage alterations can be considered of high relevance in public health. 

Previously, we conducted an epidemiologic study aimed to characterize the associations between air pollution and the frequency of different biomarkers associated with cytogenetic and genotoxic effects [22]. While higher levels of DNA adducts were detected in police officers than in controls, the frequency of micronuclei failed to show an association with air pollution exposure. Herein, we have examined in a larger cohort of traffic policemen the relationship between air pollution and epigenetic marks in the *LINE-1* and *ALU* repetitive elements and the promotor domains of the *iNOS* and the *IL-6* genes as well as the generation of DNA damage.

The personal exposure biomonitoring data showed that the concentrations of air pollutants in the city air were 7–20 folds greater among the policemen with respect to unexposed subjects. Elevated concentrations of O_3_, up to 100 µg/m^3^_,_ were also found, mainly downtown, indicating that policemen can experience exposure levels close to the European limit value of 120 µg/m^3^ O_3_ [23]. Then, one of the major findings indicated that the levels of global DNA methylation in the *ALU* repetitive elements were significantly decreased by 1.41 units in the police officers compared to unexposed clerks, after correction for confounding factors. A stronger reduction of 1.86 units was even detected among those who were controlling traffic in the downtown, a district characterized by street canyons and crowded cross-sections. Our data support the possibility that outdoor pollutants can act at epigenetic levels causing alterations of genome-wide methylation profiles in the peripheral WBCs of municipal policemen. The possibility that the decrement of global methylation in the repetitive transposable DNA elements in police officers, as seen in the current study, can be caused by road vehicle exhaust fumes is supported by extensive literary evidence where outdoor pollutants were widely associated with global DNA hypomethylation [17,24,25,26,27,28,29]. For an example, a significant reduction in global DNA methylation was reported in a cross-sectional study by Yada et al. [25] as a result of air pollution in highly polluted areas as compared to the low polluted area in India. In the investigation of De Prins et al. [24], exposure to outdoor air pollutants was significantly linked to global DNA methylation in a prospective cross-sectional study in Belgium. Hypomethylation in *LINE-1* was found by Duan et al. [26] in a cohort of coke oven workers occupationally exposed to PAHs in China. Hypomethylation in both *LINE-1* and *ALU* repeated elements was found by Tarantini et al. [27] in a cohort of workers in an electric furnace steel plant in Italy. Global hypomethylation was also linked to air pollution exposure in an Italian and a Dutch prospective cohorts of the EPIC study [28]. Previously, we have conducted an observational study to examine the changes of epigenetic marks due to occupational and environmental exposure to air pollution, as reflected by DNA methylation alterations of the *TP53*, the *cyclin-dependent kinase inhibitor 2 A* (*p16*), the *hypermethylated-in-cancer-1* (*HIC1*) and the *interleukin-6* (*IL-6*) genes and the *LINE-1* and *ALU* repetitive elements in the Ma Ta Phut industrial estate (MIE) in Rayong, Thailand [17,29]. The industrial workers of one of the largest steel, oil refinery, and petrochemical complexes in south-eastern Asia showed reduced global methylation of *LINE-1* but not with ALU in respect to rural residents with Ma Ta Phut residents exhibited methylation levels intermediate between MIE workers and rural controls. 

Subsequently, ^32^P-postlabeling results indicated that the formation of bulky DNA adducts was significantly increased by 0.45 units in policemen with respect to unexposed subjects, after adjusting for age, smoking habit, and sex. A stronger increment of 0.84 units was also observed among those who were controlling the vehicle traffic in the city centre. Exposure to tobacco smoke and environmental carcinogens can also cause oxidation reactions, which might cause formation of intra-strand cross-links between adjacent nucleotides, leading to bulky oxidative DNA modification, e.g., dimer production, detectable by ^32^P-DNA post-labelling [18,30] like other sensitive techniques of DNA damage, such the H2AX assay [31]. Our results are in line with previous epidemiology studies, which mostly reported positive associations between air pollution and DNA damage in exposed individuals, including residents in industrial areas, bus and taxi drivers, and gasoline salesmen [32]. Traffic-related air pollution in utero exposures, which could affect health later in life, were also to be linked to high levels of DNA adducts in the cordon blood of healthy pregnant women living in Copenhagen, Denmark, or resident near Superfund sites in Harris County, Texas [33]. Moreover, the levels of bulky DNA adducts have been found to be significantly correlated with air concentrations of B(a)P in occupationally and environmentally exposed workers [34]. The relationship was found to be linear at low doses and sublinear at high doses of B(a)P, suggesting that DNA adducts tend to reach a kind of saturation point at high exposure levels. 

Regression models were then used to evaluate the associations of epigenetic marks and genotoxicity with specific air pollutants. In this case, multivariable regression analysis indicated that *ALU* methylation levels of police officers were positively associated with increasing levels of external B(a)P. This result is in line with previous epigenetic studies [35,36]. Pavanello et al. [35] examined the effects of environmental exposure entailing B(a)P exposure on global DNA methylation on exposed coke-oven workers. In their study, coke-oven workers showed significantly higher DNA methylation in *ALU* and *LINE-1* repetitive elements. Both global DNA hypermethylation and hypomethylation changes were observed in human bronchial epithelial cells exposed to B(a)P, whereas no global methylation aberrations were found in human TK6 cells after treatments with different PAHs [36]. Different mechanisms can be behind epigenetic alterations due to PAH exposure. One of them can be based on the formation of BPDE at CpG islands leading to changes in the 5-mC levels or alternatively the inhibition of the activity of DNA methyltransferases [36]. The PAH-related DNA adducts can further inhibit the binding of methyl CpG binding protein 2 which is necessary to recruit DNA methyltransferases to DNA [29]. The DNA repair mechanisms can induce the substitution of methylated cytosines with native non-methylated cytosines causing methylation loss [37]. 

The risk assessment of chemical carcinogens is one of the major fields in public health as low amounts of environmental pollutants are always present and often not completely avoidable [38]. In particular, the World Health Organization (WHO) has indicated an international guideline value for the levels of O_3_ in the atmosphere of 120 µg/m^3^ [39], but an increasing number of studies have reported adverse health effects at much lower O_3_ levels [40,41,42]. Associations between premature mortality and ground-level O_3_ above 20–70 µg/m^3^ have been indicated [40,41]. A recent meta-analysis has reported an increase in birth preterm at a cut-off value of 50 µg/m^3^ O_3_ [42]. In the current study, multivariable regression analysis showed a significant global DNA hypomethylation in ALU repetitive elements above the target value at 30 µg/m3 O_3_, far below the WHO international regulations [39]. Epigenetic findings are in keeping with previous investigations reporting relationships between exposure to O_3_ and epigenetic aberrations [24,43,44,45,46,47]. For example, surface-level O_3_ exposure dependent DNA methylation changes have been found by Bind et al. [43] in a randomized crossover experiment in North Caroline, USA. In that study, suggestive changes were detected in DNA methylome of target bronchial epithelial cells after experimental O_3_ exposure relative to clean air. Reduced global DNA methylation in whole blood were associated with ground-level O_3_ in healthy adults in Belgium [24]. Ozone has been linked to decreased activity of DNA methyltransferases and hypermethylation on the *apelin* promoter in experimental rats [44]. Prenatal exposure to ground-level O_3_ has been also linked to sex-specific differential methylated regions in cord blood samples along the Early Autism Risk Longitudinal Investigation (EARLI) prospective study [45]. Surface-level O_3_ induces locus-specific hypomethylation of the *angiotensin-converting enzyme* (*ACE*), the *endothelin-1* (*ET-1*), and the *arginase* (*ARG*) genes in healthy residents in Shanghai, China [46,47]. Exposure to O_3_ has been also associated with higher urinary levels of an arachidonic metabolite, the 11-dehydro-thromboxane B2 [48], and increased expression levels of different miRNAs in human skin explants [49]. Interestingly, the threshold model indicated also an increment of bulky DNA adducts only above the target value, in agreement with previous studies [10,50], where the exposure to O_3_ was significantly associated with the formation of bulky DNA adducts in Gen-Air, a European case-control study nested in the European Prospective Investigation into Cancer and Nutrition (EPIC) project [10] and in a cross-sectional study of the Italian section of EPIC [50]. Ozone-related transformation reactions of gaseous pollutants above the cut-off value can cause the formation of different oxidised degradation products, such as the B(a)P-lactones and B(a)P-quinones [4], the mono- and diol-epoxides [51], and intermediate ozonide [52], chemicals able of directly binding to DNA to form oxidative DNA lesions.

Airborne pollutants in urban areas have progressively decreased due to preventive actions and the redesigning of motor vehicle engines. At low levels of environmental carcinogens, the most critical problem is determining the best exposure and cancer risk biomarker, allowing the integration of all the pathways and sources of exposure. Repetitive elements are representing a recognized surrogate mark for genome-wide global methylation levels their reactivation could increase genomic instability, widespread alterations in gene expression and chromatin packaging control, and disrupt gene function [53]. The DNA adducts are also considered to be a reliable biomarker of cancer risk [9,10]. Moreover, the relationship between high DNA adducts with lung cancer has been indicated by a meta-analytic approach [54]. In that meta-analysis, bulky DNA adducts were significantly associated with high lung cancer risk (95% C.I. 2.00–3.50), especially in non-smokers (95% C.I. 1.85–7.85). Taken together, our results can reflect relevant alterations of the individual “genotoxic and epigenetic tolerance” to environmental challenges caused by exposure to ground-level O_3_ above the target value of 30 µg/m^3^_._ This can be of high relevance in public health because they can cause a decline in physiological mechanisms designed to maintain cell repair and keep metabolic homeostasis, leading to tissue injury and neoplastic cell transformation [1,7]. 

Next, we found significantly higher levels of gene-specific DNA methylation in smokers compared to non-smokers; an increment of 0.73 units of the levels of *IL-6* methylation was observed in the former compared to the latter. These results confirmed that the epigenetic effects of inhalable chemicals contained in tobacco smoke can be detected in *IL-6* a multifunctional cytokine associated with signalling, such as cell differentiation and clonal selection of B cells [55,56]. The *IL-6* hypermethylation can be an epigenetic mechanism promoting *IL-6* expression in smokers, in line with early literature reports [57]. We have also shown that tobacco smoking was significantly associated with hypermethylation of the *hypermethylated-in-cancer-1* gene [29]. In that study, higher levels of methylation were detected in the promoter region of this oncogene among heavy smokers, with intermediate levels in smokers of 1–9 cigarettes/day. Instead, bulky DNA adducts tended to increase in smokers, but without reaching a statistical significance. This can reflect the kind of surrogate target, e.g., the peripheral blood, that was used in the current research. Different epidemiology studies have indeed shown that the formation of DNA damage is more easily detected in the respiratory tracts of smokers rather than in the WBCs [9,54,58]. A dose-response relationship has been indicated between the formation of bulky DNA adducts and the number of cigarettes smoked per day in the epithelium of smokers [58]. Furthermore, in a recent meta-analysis, lung cancer patients were found having with 103% greater amounts of bronchial bulky DNA adducts with respect to controls [54]. 

Our research had different major strengths. First, the enrolment of exposed subjects monitored at individual levels by personal exposure monitoring had the advantage of reducing individual-level variability of exposure assessment, which is critically important for a biomonitoring investigation. Exposure to ambient O_3_ in this observational study was measured in real-time by fixed monitoring stations offering an accurate assessment of the effects of O_3_ on the levels of biomarkers of exposed workers. Moreover, this study simultaneously evaluated the associations of ground-level O_3_ exposure with DNA methylation and DNA damage alterations in circulating peripheral blood, providing a unique opportunity to explore the molecular mechanisms behind the health effects of tropospheric O_3_. We measured blood samples obtained immediately after personal exposure monitoring, consequently reflecting recent exposure time-dependent biological processes. In comparison with other traditional DNA damage assays, such as CPD, 6-4PP ELISA, and slot blot techniques [59,60], mainly effective for DNA damage caused by exposure to ultraviolet radiation, the ^32^P-postlabeling assay has been one of the most widely applied methods in population studies for its high sensitivity to wide variety of DNA adducts and the requirement of small amounts of DNA [6]. However, our analysis may have a few limitations, such as we worked on surrogate targets, we did not analyse blood cell composition, which could influence blood DNA methylation levels [61], and we did not measure the circulating levels of the IL6 receptors along with IL6. If we had, the observed methylation differences might have emerged thanks to the high precision of the pyrosequencing technology together with the analytical method applied. However, the observed differences might indicate that a percentage of cells in each sample shows differential methylation for the considered sequences, which might have an impact on the cellular processes. It is also possible that only specific sub-populations may be differentially methylated, making, also in this case, this modification biologically relevant. Future studies should be conducted to unveil this aspect.

In summary, our study highlighted the role of air pollution exposure on epigenetic alterations and genotoxic effects. The findings suggested that exposure to air pollution above the cut-off value of 30 µg/m^3^ O_3_ can decrease global DNA methylation and elevate bulky DNA adducts levels. The magnitude of adverse molecular effects observed in the cohort of police officers underscores the necessity for developing preventative and remedial public health strategies aimed to reduce air pollution-related chronic diseases and morbidity in Western countries. Further studies with larger sample size, blood cell counts, personal monitoring of other pollutants and health outcomes measured at multiple time point are necessary to confirm our data.

## 4. Materials and Methods

### 4.1. Study Population

Police officers were recruited among a group of municipal policemen who were working in Genoa, Italy. Eligibility criteria were as follows: (1) to be employed as traffic policemen (at least for 1 year), and (2) to have no reported history of occupational carcinogen exposures. Age-matched healthy unexposed subjects were selected at San Martino Hospital, Genoa, Italy among office clerks by local health service. Unexposed subjects included subjects without a history of occupational and environmental exposure to carcinogens. The study was conducted in accordance with the guidelines of the Helsinki Declaration and approved by the Institutional Review Board of San Martino Hospital (n. EA97001, 02-02-97). All the participants were informed about the aims of the study and provided written informed consent. All the study volunteers performed their personal sampling during their work shift and the blood samples along with the questionnaires were gathered at the end of the shift. Details about age, gender, professions, life-style habit, occupational status, and history were obtained by means of a detailed questionnaire. 

### 4.2. Air Pollution Exposure Analysis

Personal exposure biomonitoring was used to analyse the airborne levels of B(a)P (ng/m^3^) and carbon monoxide CO (mg/m^3^) in the breathing zone of the study population, as previously described [22]. Participants were monitored for 4 h/day by battery-operated personal devices. Ground-level O_3_ (µg/m^3^) was obtained by fixed monitoring stations located in three areas of Genoa, Italy, e.g., hill and seaside suburbs and downtown. 

### 4.3. Bulky DNA Adduct Analysis 

Bulky DNA adducts were analysed blindly by the ^32^P-DNA post-labelling assay [18], using a chromatographic system able to detect B(a)P-, lactone- and quinone-DNA adducts and bulky oxidative DNA lesions [4,20]. Detection and quantification of DNA adducts were performed by storage phosphor imaging employing intensifying screens. Intensifying screens were scanned using a Typhoon 9210 (Amersham) and the software used to process the data was ImageQuant (version 5.0) from Molecular Dynamics. The DNA adducts were expressed as relative adduct labelling (RAL) × 10^8^ normal nucleotides = pixels in adducted nucleotides/pixels in normal nucleotides. The bulky DNA adducts’ levels were corrected across experiments based on the recovery of two carcinogen-adducted DNA standards: one standard was prepared by in vitro experiments through the reaction of B(a)P diolepoxide (BPDE, ^3^H-labelled) with human DNA [19]. The other carcinogen-adducted DNA standard consisted of a DNA sample extracted from the hepatic tissue of experimental mice i.p. treated for 24 h with 1.0 mg of [7,8-^3^H]B(a)P. The concentrations of B(a)P adducts in the livers of the B(a)P treated mice were also quantified as B(a)P-tetrols released from hydrolysis of macromolecules and measured by gas-chromatography-mass spectrometry (GC-MS) methods [19].

### 4.4. DNA Methylation Analysis

The DNA methylation was quantified using bisulfite-PCR and pyrosequencing [17]. In detail, a 50 μL PCR reaction was carried out with 25 μL of Hot Start GoTaq Green Master mix, 1 pmol of forward primer, 1 pmol of biotinylated reverse primer, and 25 ng of bisulfite-treated genomic DNA. Biotin-labeled primers were used to purify the final PCR product with sepharose beads. The PCR product was bound to a Streptavidin Sepharose HP. Sepharose beads containing the immobilized PCR product were purified, washed, denatured with 0.2 M NaOH, and washed again with the Pyrosequencing Vacuum Prep Tool according to the manufacturer’s instructions. The pyrosequencing primer (0.3 μM) was annealed to the purified single-stranded PCR product, and pyrosequencing was performed with the PyroMark MD System. The CpG sites were interrogated in the promoter domains of iNOS gene and within the repetitive elements LINE-1 and ALU. Primer sequences are reported in Table 5. The DNA methylation at CpG positions within the repetitive elements and each gene’s promoter region were expressed as the percentage of cytosines that were methylated, determined as the number of 5mC divided by the sum of methylated and unmethylated cytosines, multiplied by 100% (%5mC). Every DNA sample was tested twice to confirm reproducibility and increase the precision of the findings.

### 4.5. Statistical Analysis

Standard descriptive analyses were used to evaluate the levels of global and specific-DNA methylation and the formation of bulky DNA adducts in the WBCs of the study population according to smoking habit, exposure status, working districts, and other study variables. Multivariate analyses were performed by fitting linear regression models adjusted by age (continuous), gender, and smoking habit (smokers and non-smokers). Means Difference (MD) and 95% Confidence Intervals (C.I.) were used to evaluate the effect of each level of predictor variables with respect to its reference level and to estimate the relationship of DNA methylation and bulky DNA adduct biomarker with air pollution and specific pollutants. A *p*-value of 0.05 was considered statistically significant. Data were analysed using SAS9.3 and SPSS 20.0 (IBM SPSS Statistics, New York, NY, USA).

## Figures and Tables

**Table 1:** Concentrations of benzo(a)pyrene [B(a)P] ng/m^3^, carbon monoxide (CO mg/m^3^), and ground-level ozone (O_3_) µg/m^3^ by personal exposure monitoring or local monitoring stations in the city air of Genoa, Italy. Air pollutants are also reported according to working districts (seaside, hill, and downtown).

Traffic-Related Air Pollution							
		B(a)P ng/m^3^		CO mg/m^3^		O_3_ µg /m^3^	
	*n*	Mean ± SD		Mean ± SD		Mean ± SD	
Status							
Unexposed subjects	45	0.2 ± 0.3		1.1 ± 0.4		-	
Policemen	95	4.2 ± 2.6	<0.001	8.7 ± 4.2	<0.001	44.2 ± 23.5	
Districts							
Seaside	21	5.2 ± 2.2		8.2 ± 4.5		28.2 ± 9.9	
Hill	22	3.4 ± 3.1	0.050	8.7 ± 3.4	0.704	50.5 ± 17.7	<0.001
Downtown	52	4.3 ± 2.5	0.221	9.0 ± 4.4	0.503	47.4 ± 26.7	0.003

**Table 2:** Average levels of DNA methylation in the repetitive elements of the *Long Interspersed Nuclear Element-1* (*LINE-1*) and *ALU*, and in the promoter domains of the *interleukin-6* (*IL-6)* and the *inducible nitric oxide synthase* (i*NOS)* genes, expressed as a percentage of 5-methylcytosine, and the mean frequency of bulky DNA adducts, expressed as relative adduct labelling × 10^8^ normal nucleotides, according to the smoking habit, exposure status and three working suburbs of Genoa, Italy (seaside, hill, and downtown).

Epigenetic and DNA Damage Marks						
		*LINE-1*	*ALU*	*IL-6*	*INOs*	DNA Adducts
	*n*	Mean ± SD	Mean ± SD	Mean ± SD	Mean ± SD	Mean ± SD
Smoking habit						
Non-smokers	53	73.4 ± 1.0	30.1 ± 2.7	5.0 ± 1.4	64.4 ± 4.2	1.2 ± 1.1
Smokers	97	73.4 ± 1.4	29.6 ± 3.5	5.6 ± 2.4	63.6 ± 5.2	1.4 ± 1.2
Occupational status						
Unexposed subjects	45	73.4 ± 1.1	31.1 ± 3.4	5.2 ± 2.5	64.0 ± 5.2	0.9 ± 0.6
Policemen	95	73.4 ± 1.2	29.3 ± 3.2	5.1 ± 1.4	64.4 ± 4.3	1.4 ± 1.3
Working districts						
Seaside	21	73.2 ± 1.6	30.9 ± 3.9	5.1 ± 1.4	64.2 ± 4.1	0.9 ± 0.9
Hill	22	73.4 ± 1.1	29.5 ± 2.6	4.8 ± 1.4	63.9 ± 4.4	0.9 ± 0.8
Downtown	52	73.6 ± 1.0	28.8 ± 3.0	5.3 ± 1.4	64.5 ± 4.4	1.8 ± 1.5

**Table 3:** Means Difference (MD) with respect to reference level of each variable and 95% Confidence Intervals (C.I.) of DNA methylation in the repetitive elements of the *Long *Interspersed* Nuclear Element-1* (*LINE-1*) and *ALU* and in the promoter domains of the *interleukin-6* (*IL-6)* and the *inducible nitric oxide synthase* (i*NOS)* genes and MD and 95% C.I. of bulky DNA adducts according to smoking habit, exposure status, and three working suburbs of Genoa, Italy (seaside, hill, and downtown).

Epigenetic and DNA Damage Marks										
	*LINE-1*		*ALU*		*IL-6*		*INOs*		DNA Adducts	
	MD 95% C.I.	*p*	MD 95% C.I.	*p*	MD 95% C.I.	*p*	MD 95% C.I.	*p*	MD 95% C.I.	*p*
Status										
Smokers	0.01 −0.39–0.42	0.942	−0.19 −1.39–1.00	0.750	0.73 0.07–1.39	0.030	−0.74 −2.46–0.98	0.395	0.07 −0.35–0.48	0.756
Policemen	0.04 −0.38–0.46	0.857	−1.41 −2.65–−0.17	0.026	−0.26, −0.94–0.42	0.447	0.50 −1.28–2.28	0.578	0.45 0.02–0.88	0.039
Working districts										
Seaside	−0.11 −0.74–0.53	0.734	0.48 −1.36–2.32	0.603	−0.33 −1.36–0.71	0.530	0.01 −2.69–2.72	0.993	−0.19 −0.81–0.42	0.530
Hill	0.10 −0.52–0.73	0.746	−0.73 −2.54–1.09	0.430	−0.71 −1.71–0.29	0.161	0.46 −2.15–3.07	0.728	−0.18 −0.78–0.43	0.563
Downtown	0.16 −0.32–0.63	0.511	−1.86 −3.23–−0.49	0.008	−0.13 −0.89–0.63	0.735	0.39 −1.62–2.39	0.704	0.84 0.39–1.29	<0.001

**Table 4:** Means Difference (MD) and 95% Confidence Intervals (C.I.) of DNA methylation in the repetitive elements of the *Long Interspersed Nuclear Element-1*(*LINE-1*) and *ALU*, and in promoter domains of the *interleukin-6* (*IL-6)* and *inducible nitric oxide synthase* (i*NOS)* genes, and MD and 95% C.I. of bulky DNA adducts according to the concentrations of benzo(a)pyrene [B(a)P ng/m^3^], carbon monoxide (CO mg/m^3^) and ground-level ozone (O_3_ µg/m^3^). MD and 95% C.I. of biomarkers were also examined using a cut-off value of 30 µg/m^3^ O_3_.

Epigenetic and DNA Damage Marks										
	*LINE-1*		*ALU*		*IL-6*		*INOs*		DNA Adducts	
	MD 95% C.I.	*p*	MD 95% C.I.	*p*	MD 95% C.I.	*p*	MD 95% C.I.	*p*	MD 95% C.I.	*p*
External B(a)P levels (ng/m^3^)	−0.01 −0.11–0.08	0.790	0.32 0.03–0.60	0.032	0.07 −0.05–0.19	0.282	−0.13 −0.50–0.24	0.485	−0.10 −0.21–0.01	0.052
External CO levels (mg/m^3^)	0.04 −0.02–0.10	0.186	0.06 −0.12–0.25	0.510	0.01 −0.07–0.08	0.856	−0.06 −0.29–0.18	0.626	−0.02 −0.09–0.04	0.488
Ground-level O_3_ and cut-off value										
<30 µg/m^3^ cut-off	−0.11 −0.64–0.43	0.694	−0.96 −2.53–0.60	0.226	−0.27 −1.13–0.58	0.530	0.71 −1.54–2.96	0.532	0.26 −0.28–0.80	0.342
≥30 µg/m^3^ cut-off	0.12 −0.34–0.58	0.609	−1.65 −3.01–−0.30	0.017	−0.26 −1.00–0.49	0.499	0.38 −1.56–2.33	0.698	0.56 0.09–1.03	0.019

**Table 5:** Primers for DNA methylation in the repetitive elements of *LINE-1* (*Long Interspersed Nuclear Element-1*) and *ALU*, and in the promoter domains of the *interleukin-6* (*IL-6)* and the *inducible nitric oxide synthase* (i*NOS)* genes.

	ID Forward Primer (5′-3′)	Reverse Primer (5′-3′)	Sequencing Primer (5′-3′)
*LINE-1*	TTTTGAGTTAGGTGTGGGATATA	Biotin-AAAATCAAAAAATTCCCTTTC	AGTTAGGTGTGGGATATAGT
*ALU*	biotin-TTTTTATTAAAAATATAAAAATT	CCCAAACTAAAATACAATAA	AATAACTAAAATTACAAAC
*INOs*	AATGAGAGTTGTTGT TGGGAAGTGTTT	Biotin-CCACCAAACCCAACCAAACT	TAAAGGTATTTTTGTTTTAA
*IL-6*	biotin-TATTTTAGTTTTGAGAAAGGAGGTG	CAATACTCTAAAACCCAACAAAAAC	TCCTAATACAAACAACCCC

## Data Availability

Data are available upon reasonable request.
